# Identifying the Relationship Structure Among Multiple Datasets Using Independent Vector Analysis: Application to Multi-Task fMRI Data

**DOI:** 10.1109/access.2024.3435526

**Published:** 2024-07-29

**Authors:** ISABELL LEHMANN, TANUJ HASIJA, BEN GABRIELSON, MOHAMMAD A. B. S. AKHONDA, VINCE D. CALHOUN, TÜLAY ADALI

**Affiliations:** 1 Signal and System Theory Group, Paderborn University, 33098 Paderborn, Germany; 2 Department of Computer Science and Electrical Engineering, University of Maryland, Baltimore County (UMBC), Baltimore, MD 21250, USA; 3 Tri-Institutional Center for Translational Research in Neuroimaging and Data Science (TReNDS), Georgia State University, Georgia Institute of Technology, and Emory University, Atlanta, GA 30303, USA

**Keywords:** Blind source separation, bootstrap, data-driven, fMRI, independent vector analysis, relationship structure

## Abstract

Identifying relationships among multiple datasets is an effective way to summarize information and has been growing in importance. In this paper, we propose a robust 3-step method for identifying the relationship structure among multiple datasets based on Independent Vector Analysis (IVA) and bootstrap-based hypothesis testing. Unlike previous approaches, our theory-backed method eliminates the need for user-defined thresholds and can effectively handle non-Gaussian data. It achieves this by incorporating higher-order statistics through IVA and employing an eigenvalue decomposition-based feature extraction approach without distributional assumptions. This way, our method estimates more interpretable components and effectively identifies the relationship structure using hierarchical clustering. Simulation results demonstrate the effectiveness of our method, as it achieves perfect Adjusted Mutual Information (AMI) for different values of the correlation between the components. When applied to multi-task fMRI data from patients with schizophrenia and healthy controls, our method successfully reveals activated brain regions associated with the disorder, and identifies the relationship structure of task datasets that matches our prior knowledge of the experiment. Moreover, our proposed method extends beyond task datasets, offering broad applicability in subgroup identification in neuroimaging and other domains.

## I. INTRODUCTION

In recent years, identifying relationships among multiple datasets has received growing attention in medical applications, such as for making group inferences when estimating brain activations [1] or for better localization of brain activity [2]. By defining a *grouping* as a subset of datasets that share some latent characteristics, analyzing how the datasets are distributed in these groupings and how these groupings are related reveals the structure of these relationships. Identifying this *relationship structure* provides an even finer understanding of the complete dataset and therefore goes beyond only simple identification of the groupings.

One potential application of this is subgroup identification [3]. In this context, each dataset corresponds to a subject, and a grouping of subject datasets is called a subject grouping or *subgroup*. The identified subgroups can be used in multiple applications. For example, in Electronic Health Records (EHR) data, identifying subgroups can help uncover previously unknown connections between illnesses [4], while in precision medicine, estimating the dose of medication for a patient can be achieved based on the known dose of other patients in the same subgroup [5], [6]. Furthermore, by identifying the relationship structure among the datasets in a mixed cohort, for example, the associations among data of patients with schizophrenia, bipolar disorder, and their subtypes can be found.

There are multiple approaches for subgroup identification For example, in [7], a subgroup identification method is proposed that allows the inclusion of any set of covariates for classification, while in [8], model-based partitioning is introduced for estimating a treatment effect function to identify subgroups that are linked to predictive factors through a decision tree. Many of these subgroup identification methods are either model-based [8] or heuristic approaches [7], and thus have strong assumptions or can be prone to subjective thresholds. Furthermore, they all are only identifying the subgroups, but not the relationship among them. These limitations highlight the need for further development and refinement of methods that can effectively learn the relationship structure among multiple datasets data without subjective choices. Here, data-driven techniques provide an alternative approach to the problem by extracting lower-dimensional features linked across multiple datasets. Joint Blind Source Separation (JBSS) techniques, especially those based on matrix decompositions such as Independent Component Analysis (ICA), summarize multiple datasets through latent variables, called *sources/components*, that are directly interpretable, without imposing strong constraints [9]. Based on these latent components, *subgroups* can be identified as subsets of datasets where the components within a subgroup are more similar to each other than to those outside the subgroup.

The well-known matrix decomposition method ICA is based on a linear mixture model, where a dataset is decomposed into a mixing matrix and sources, which are assumed to be statistically independent. ICA has been found to be successful for decomposing neuroimaging datasets, e.g., functional Magnetic Resonance Imaging (fMRI) datasets, where usually little is known about the underlying sources [9], [10]. ICA has been extended to analyze multi-set data using either the joint ICA [11] or the group ICA [10] models, which are limited in performance, though, as they assume a common mixing matrix or source vector for all datasets. Independent Vector Analysis (IVA) is a more flexible extension of ICA to multiple datasets that makes use of the statistical dependence of the source components across datasets [9], [12]. Over the years, there has been an increase in the utilization of IVA for fMRI data fusion [13], [14] because IVA 1) naturally aligns the sources across datasets, 2) effectively retains subject variability in multi-subject fMRI data [15], and 3) can identify common and distinct brain activations between groups of patients and controls [13], [14], [16].

In [13], subgroup identification is performed using IVA, where subgroups of patients with schizophrenia are found with a semi-heuristic procedure with the goal of better understanding the underlying heterogeneity of schizophrenia. An approach based on IVA and Gershgorin discs is used for subgroup identification in [16], but their method identifies subgroups within each Source Component Vector (SCV) separately, which is less robust compared with taking the information from several SCVs together into account. A more comprehensive review of data-driven subgroup identification methods can be found in [17]. Furthermore, these approaches only identify the subgroups, but do not reveal their full relationship structure. For example, in [18], hierarchical clustering is used for identifying groupings of representative rules that are used for subgroup discovery instead of directly revealing the relationship among different datasets. Finally, the authors of [14] show that information about the relationship of the datasets can be visually seen in the covariance matrices of the SCVs estimated by IVA, but they do not provide an objective method for fusing the information of all SCVs together to discover the full relationship structure. Herein lies the novelty of our paper: we do not only discover groupings of the datasets but also identify their relationship structure. Moreover, we leverage information from all SCVs collectively, compared with considering each SCV individually, which enhances the robustness of our method.

More precisely, in this paper, we propose a powerful method for identifying the relationship structure among multiple datasets by leveraging key properties of IVA. To the best of our knowledge, no other method exists to achieve this goal. In our 3-step method, 1) a latent representation of the datasets is found using IVA, 2) the SCVs are identified that contain information about the relationship of the datasets, and 3) features are extracted from these SCVs as the input of a hierarchical clustering algorithm. By finding how close these datasets are to each other, our method does not only identify groupings of the datasets but also fully discovers their relationship structure in a resulting dendrogram. We summarize the contributions of this work as follows:

- developing a method for identifying the relationship structure among multiple datasets,
- verifying the success of our method by identifying a meaningful relationship structure in multi-task fMRI datasets, as the method groups together the task datasets from similar tasks, and
- revealing interpretable latent components in the fMRI data and observing significantly stronger deactivation of the Default Mode Network (DMN) areas in patients with schizophrenia compared with healthy controls.

With simulated datasets, we experimentally demonstrate the superior performance of our proposed method compared with the competing techniques. Our paper is organized as follows: We formulate the problem in section II and explain our method for solving it in section III. Then, we evaluate our method in simulations in section IV, apply it to real multi-task fMRI data in section V, and finally summarize and discuss the main findings of our paper in section VI.

## II. PROBLEM FORMULATION

Let $x^{\left[k\right]}\in {\mathbb{R}}^{R}$ denote the $k^{\text{th}}$ dataset, where R denotes the dimension of the dataset.

Given K datasets, identify their relationship structure, i.e., group datasets based on the similarity (high statistical dependence) between their latent variables. Note that this cannot be achieved through simple clustering approaches, as revealing the relationship structure relies on the dependence of the latent variables across datasets.

The datasets are assumed to be generated according to the following model:

(1) $$x^{\left[k\right]}=A^{\left[k\right]}s^{\left[k\right]},\;k=1,\ldots ,K$$

where $s^{\left[k\right]}\in {\mathbb{R}}^{R}$ is the latent source vector containing R source components, and $A^{\left[k\right]}\in {\mathbb{R}}^{R\times R}$ is the unknown mixing matrix. For example, in fMRI data, R can correspond to the number of subjects, and the sources are spatial maps.

Let the $r^{\text{th}}$ Source Component Vector (SCV) be defined as the concatenation of the $r^{\text{th}}$ source component of all K datasets [9]:

(2) $$s_{r}={\left[\begin{array}{c} s_{r}^{\left[1\right]}\ldots s_{r}^{\left[K\right]} \end{array}\right]}^{⊤}\in {\mathbb{R}}^{K},$$

where $s_{r}^{\left[k\right]}\in \mathbb{R}$ denotes the $r^{\text{th}}$ source component of the $k^{\text{th}}$ dataset. Without loss of generality, the source components are assumed to be zero-mean and unit-variance. Furthermore, we assume the source components to be independent among SCVs, which is a common assumption for identifying source components by only observing datasets [19]. This way, the definition of an SCV allows to capture the dependence information across multiple datasets within an SCV. As the source components are zero-mean and unit-variance, the covariance matrix of the $r^{\text{th}}SCV$ coincides with its correlation matrix and can be defined as

(3) $$C_{r}=E\left[s_{r}s_{r}^{⊤}\right]\in {\mathbb{R}}^{K\times K}.$$

In [13] and [14], it is shown that information about the relationship of the datasets can be inferred from the linear dependence (correlation) of the source components across datasets. This information is revealed in the covariance matrices of the SCVs. An SCV covariance matrix with all non-zero off-diagonal values implies that all components within that SCV are dependent, and hence describes a *common* SCV, i.e., with the components common across all datasets [13]. Similarly, an SCV covariance matrix exhibiting a block-diagonal structure (with off-block elements equal to 0) implies that all components within each block are dependent. We call SCVs with these covariance matrices *structured*. Note that this also holds for covariance matrices that can be transformed into block-diagonal matrices using an orthogonal permutation matrix $P\in {\mathbb{R}}^{K\times K}$ s.t. ${\tilde{C}}_{r}={PC}_{r}P^{⊤}$ is block-diagonal. While common SCVs only provide limited useful information about the relationship between the datasets, structured SCVs are most informative for this. Consequently, in this paper, we are primarily interested in identifying the structured SCVs and then using them for identifying the relationship structure of the datasets.

## III. METHOD

**FIGURE 1.**
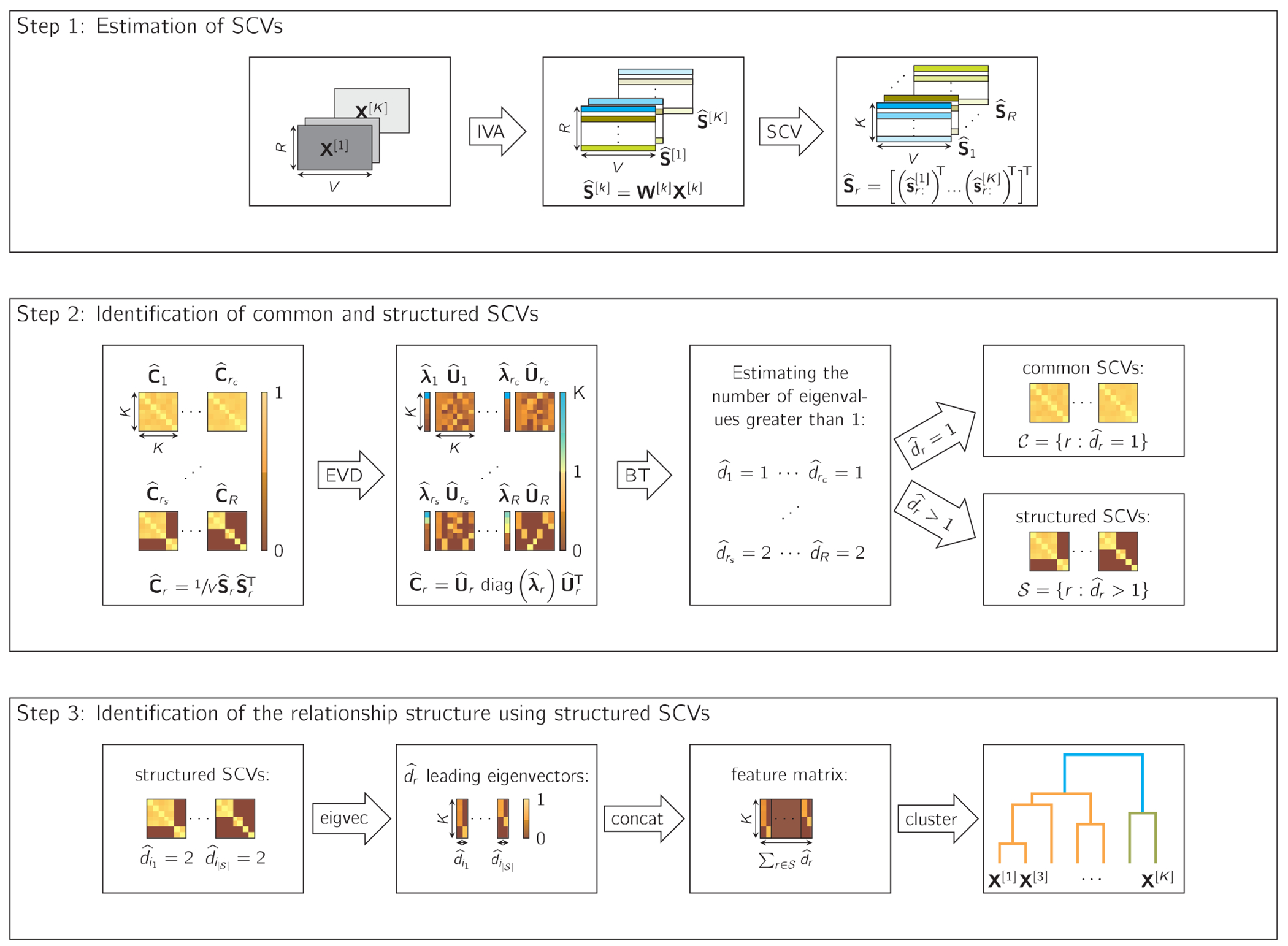
Visualization of our proposed method for identifying the relationship structure among multiple datasets. We have K observed datasets of dimension R×V, where V is the number of voxels and R the (reduced) dimension of the data. In step 1, the latent sources in the SCVs ${\hat{S}}_{r}$ are estimated by applying IVA on the observed datasets. In step 2, the SCVs are identified as structured or common by applying an eigenvalue decomposition (EVD) on their covariance matrices and using the proposed bootstrap technique (BT) to estimate ${\hat{d}}_{r}$, i.e., the number of eigenvalues greater than 1. If ${\overset{ˆ}{d}}_{r}=1$, the $r^{\text{th}}$ SCV is identified as common, and if ${\overset{ˆ}{d}}_{r}>1$, as structured. We denote the indices of the structured SCVs as $𝒮=\left\{i_{1},\ldots ,i_{|𝒮|}\right\}$. In Step 3, the ${\overset{ˆ}{d}}_{r}$ leading eigenvectors of the structured SCVs are concatenated to form a feature matrix, which is the input of hierarchical clustering. The resulting clusters are the identified groupings (in this example, there are two groupings: orange and green), and the dendrogram reveals the relationship structure among the datasets.

In this paper, we propose a method for identifying the relationship structure among multiple datasets. Figure 1 shows the three steps of which our method consists: 1) estimation of latent sources, 2) identification of common and structured SCVs, and 3) identification of the relationship structure using structured SCVs. The following sections explain the details of each step.

### A. STEP 1: ESTIMATION OF SCVS

To be able to infer information about the relationship structure from the SCV covariance matrices, the unknown source components in the SCVs must be estimated from the observed datasets. For this, we use IVA.

#### 1) INDEPENDENT VECTOR ANALYSIS

The generative model for IVA is given by (1). The goal of IVA is to jointly estimate the K source vectors [12]

(4) $${\hat{s}}^{\left[k\right]}=W^{\left[k\right]}x^{\left[k\right]},$$

where ${\hat{s}}^{\left[k\right]}\in {\mathbb{R}}^{R}$ denotes the estimate of $s^{\left[k\right]}$, and $W^{\left[k\right]}\in {\mathbb{R}}^{R\times R}$ is the demixing matrix for the $k^{\text{th}}$ observed dataset. The degree of independence is measured by mutual information among the estimated SCVs, defined as [9]

(5) $$I\left({\hat{s}}_{1};\ldots ;{\hat{s}}_{R}\right)=\sum_{r=1}^{R}\left(\sum_{k=1}^{K}H\left({\hat{s}}_{r}^{\left[k\right]}\right)-I\left({\hat{s}}_{r}\right)\right)-\sum_{k=1}^{K}\text{log}\left|\text{det}\left(W^{\left[k\right]}\right)\right|-C,$$

where $I\left({\hat{s}}_{r}\right)$ is the mutual information of the K source components in the $r^{\text{th}}$ estimated SCV ${\hat{s}}_{r},H\left({\hat{s}}_{r}^{\left[k\right]}\right)$ denotes the entropy of ${\hat{s}}_{r}^{\left[k\right]}$, and C is a constant term. By minimizing the mutual information among the estimated SCVs, IVA maximizes independence among the SCVs, while simultaneously maximizing the mutual information within each SCV. Through the selection of an appropriate multivariate density model, IVA can take either second-order or all-order statistics into account [9]. IVA can correctly estimate the SCVs if and only if there exist no subsets of source components within two SCVs that meet the following conditions: 1) the source components in the subsets are Gaussian distributed and independent of the other source components within the same SCV, and 2) the source components in the subsets of the two SCVs have proportional covariance matrices [9]. Hence, IVA is able to identify a very broad class of signals.

IVA-L-SOS [15] assumes that the SCVs underlay a multivariate Laplacian distribution while allowing for a non-identity covariance matrix. This way, dependence is not only measured by higher-order statistics, but correlations, i.e., second-order statistics, are also taken into account. As it has been shown that the modeling assumptions of IVA-L-SOS are a good match for the properties of real fMRI data [13], [15], we will use it in our method. However, if our method is applied to data with different source distributions, Step 1 can be replaced by another source separation method with appropriate assumptions for that type of data.

#### 2) SAMPLES

In practice, we observe V samples of each dataset $x^{\left[k\right]}$, which form the observed datasets $X^{\left[k\right]}\in {\mathbb{R}}^{R\times V}$. Then, the estimated source matrices are

(6) $${\hat{S}}^{\left[k\right]}=W^{\left[k\right]}X^{\left[k\right]}\in {\mathbb{R}}^{R\times V},$$

and the $r^{\text{th}}$ estimated SCV is defined as

(7) $${\hat{S}}_{r}={\left[\begin{array}{lll} {\left({\hat{s}}_{r:}^{\left[1\right]}\right)}^{⊤} & \ldots & {\left({\hat{s}}_{r:}^{\left[K\right]}\right)}^{⊤} \end{array}\right]}^{⊤}\in {\mathbb{R}}^{K\times V},$$

where ${\hat{s}}_{r:}^{\left[k\right]}\in {\mathbb{R}}^{1\times V}$ denotes the $r^{th}$ row of ${\hat{S}}^{\left[k\right]}$, i.e., the $r^{th}$ estimated source component of the $k^{\text{th}}$ dataset. The estimation of the SCVs with samples is shown in Step 1 in Figure 1.

### B. STEP 2: IDENTIFICATION OF COMMON AND STRUCTURED SCVS

In Step 2, we must determine which of the estimated SCVs are common and which are structured. Only the structured SCVs contain information about the relationship between the datasets, and thus, only these structured SCVs are utilized in Step 3 to determine features for identifying the relationship structure.

The SCVs estimated by IVA-L-SOS can be identified as either common or structured based on the eigenvalues of their covariance matrices. Let the Eigenvalue Decomposition (EVD) of the true covariance matrix of the $r^{\text{th}}$ SCV $C_{r}$ (defined in (3)) be

(8) $$C_{r}=U_{r}\;diag\left(\lambda_{r}\right)U_{r}^{⊤}$$

where $U_{r}\in {\mathbb{R}}^{K\times K}$ contains the eigenvectors of $C_{r}$ as columns, and $\lambda_{r}\in {\mathbb{R}}^{K}$ contains the corresponding eigenvalues. When $C_{r}=I$, where I denotes the identity matrix, all its eigenvalues are equal to 1. However, when $C_{r}\neq I$, some eigenvalues are different from 1. Let $d_{r}$ be defined as the number of eigenvalues of $C_{r}$ that are greater than 1. We assume that the SCV covariance matrices have only one of the following two structures:

- For *common* SCVs, where all the source components within an SCV are correlated with each other, the covariance matrix $C_{r}$ has 1s on the diagonal, and all off-diagonal values (corresponding to the correlation coefficients) are non-zero. In [20], it is shown that, under certain conditions on correlation coefficients, $C_{r}$ has exactly one eigenvalue greater than 1, i.e., $d_{r}=1$.
- For *structured* SCVs, the covariance matrix $C_{r}$ is block-diagonal with 1s on the diagonal and zero off-block values (or can be permuted into a block-diagonal matrix ${\tilde{C}}_{r}=PC_{r}P^{⊤}$, where $P\in {\mathbb{R}}^{K\times K}$ is an orthogonal permutation matrix). We assume that the covariance matrices of structured SCVs have at least two blocks. To determine the number of eigenvalues greater than 1 for such SCV covariance matrices, we present the following corollary:
  *Corollary 1: Let* $C_{r}\in {\mathbb{R}}^{K\times K}$ *be a block-diagonal matrix consisting of* $d_{r}$ *blocks* $B_{i}\in {\mathbb{R}}^{K_{i}\times K_{i}}$ *such that*
$$C_{r}=\left[\begin{array}{cccc} B_{1} & 0 & \ldots & 0\\ 0 & \ddots & \ddots & \vdots \\ \vdots & \ddots & B_{d_{r}} & 0\\ 0 & \ldots & 0 & I \end{array}\right],$$
  *and* $B_{i}$ *contains 1s on the diagonal and positive entries on its off-diagonal elements. Then*, $C_{r}$ *has exactly* $d_{r}$ *eigenvalues greater than one*.
  As it is proven in [20] that $\left|\left\{\lambda \left(B_{i}\right)>1\right\}\right|=1$ (and the remaining eigenvalues are less than or equal to 1), it follows naturally for $d_{r}$ blocks in $C_{r}$ that $\left|\left\{\lambda \left(C_{r}\right)>1\right\}\right|=d_{r}$. Note that Corollary 1 also holds if ${\tilde{C}}_{r}$ (and not $C_{r}$) is block-diagonal because ${\tilde{C}}_{r}$ has the same eigenvalues as $C_{r}$.

Under the assumption that a structured SCV consists of at least two blocks, we can differentiate between a common SCV $\left(d_{r}=1\right)$ and a structured SCV $\left(d_{r}>1\right)$ by counting the number of eigenvalues greater than 1. We define $𝒞=\left\{r\;:\;d_{r}=1\right\}$ as the set of indices of the common SCVs and $𝒮=\left\{r\;:\;d_{r}>1\right\}$ as the set of indices of the structured SCVs. In the following, we denote the indices in 𝒮 as

(9) $$𝒮=\left\{i_{1},\ldots ,i_{\left|𝒮\right|}\right\}.$$

Note that we do not consider SCVs with identity covariance matrices, which would correspond to completely uncorrelated datasets, because we assume that in real data some correlations typically exist in each SCV.

Now, when we estimate covariance matrices over finite samples V,

(10) $${\hat{C}}_{r}=\frac{1}{V}{\hat{S}}_{r}{\hat{S}}_{r}^{⊤},$$

the estimated correlation coefficients corresponding to the uncorrelated datasets will not be exactly zero, and thus, more than $d_{r}$ eigenvalues for a structured SCV will be greater than 1. Thus, by just counting the eigenvalues greater than 1, $d_{r}$ would be overestimated. Consequently, it is necessary to estimate $d_{r}$. Estimating the number of significant eigenvalues is commonly addressed as a model-order selection problem in the literature [21]. However, these model-order selection techniques assume certain asymptotic properties (as V→∞) on the non-significant eigenvalues, for example, assuming they all or a subset of them are equal to each other (in [20] and [21]). This is not applicable for the SCV covariance matrices. For example, if all the source components in an SCV covariance matrix would belong to one of the blocks, and thus there would be no uncorrelated source components, then for arbitrary correlation coefficients, none of the non-significant eigenvalues would be equal to each other.

To estimate $d_{r}$ for the $r^{\text{th}}$ SCV covariance matrix $C_{r}$, we perform a binary hypothesis test for each k=1,…,K with the null hypothesis

(11) $$H_{0}\;:\;\lambda_{r}^{\left[k\right]}\leq 1,$$

and the alternative

$$H_{1}\;:\;\lambda_{r}^{\left[k\right]}>1.$$

Here, $\lambda_{r}^{\left[k\right]}$ is the $k^{\text{th}}$ eigenvalue of $C_{r}$. As in practice we only estimate ${\hat{C}}_{r}$, we only know the estimated eigenvalues ${\hat{\lambda }}_{r}^{\left[k\right]}$. We define a test statistic $\hat{T}={\hat{\lambda }}_{r}^{\left[k\right]}-1$, and to perform the hypothesis test, we must know the distribution of the statistic under $H_{0}$ [22]. Neither the sample nor the asymptotic distribution of the test statistic is known. We, therefore, propose a bootstrap-based hypothesis test [23] to estimate this distribution. Under certain conditions, the distribution estimated by bootstrap converges to the true distribution if the number of samples goes to infinity (V→∞) [24], and thereby we can estimate ${\hat{d}}_{r}$ for each SCV.

The pseudocode for our method is described in Algorithm 1. In the following, we describe the steps of the algorithm.

1. The sample correlation matrix ${\hat{C}}_{r}=\left|\frac{1}{V}{\hat{S}}_{r}{\hat{S}}_{r}^{⊤}\right|$ is calculated (line 1). The absolute value is necessary because of a possible sign ambiguity in the sources. Then, an EVD is applied on ${\hat{C}}_{r}$ to get the eigenvalues ${\hat{\lambda }}_{r}=\left[{\overset{ˆ}{\lambda }}_{r}^{\left[1\right]},\ldots ,{\hat{\lambda }}_{r}^{\left[K\right]}\right]$, sorted in descending order (line 2). Here, ${\hat{\lambda }}_{r}^{\left[k\right]}$ denotes the estimate of $\lambda_{r}^{\left[k\right]}$.

**Algorithm 1:** Bootstrap Algorithm for Estimating ${\hat{d}}_{r}$

**Input:** ${\hat{S}}_{r}\in {\mathbb{R}}^{K\times V}$, *B*, *P*_fa_
1: ${\hat{C}}_{r}\leftarrow abs\left(\frac{1}{V}{\hat{S}}_{r}{\hat{S}}_{r}^{⊤}\right)$
2: ${\hat{\lambda }}_{r},{\hat{U}}_{r}\leftarrow \mathrm{EVD}\left({\hat{C}}_{r}\right)\;\;\;\;\;\;▷\;\text{s.t.}\;{\hat{\lambda }}_{r}^{\left[1\right]}\geq \cdots \geq {\hat{\lambda }}_{r}^{\left[K\right]}$
3: **for** *b* = 1, …, *B* **do**
4: _*b*_**J** ← randint(1, *V*, *V*) $\;▷\;V\;\text{integers}∼{𝒰}_{\text{int}}(1,V)$
5: S^rb←S^r(:,bj)
6: C^rb←abs(1VS^rbS^r⊤b)
7: λ^rb,U^rb←EVD(C^rb)
8: **end for**
9: **for** k=1,…,Kdo
10: $\hat{T}\leftarrow {\hat{\lambda }}_{r}^{\left[k\right]}-1$
11: **for** b=1,…,Bdo
12: T^*b←bλ^r[k]−λ^r[k]
13: **end for**
14: T^∗←sort(T^∗)▷s.t.T^*1≤⋯≤T^*B
15: $\eta =\lfloor (B+1)\cdot \left(1-P_{\text{fa}})\right\rfloor$
16: **if** T^<T*ηthen
17: $\epsilon^{\left[k\right]}\leftarrow 0\;\;\;\;\;\;\;\;\;▷\;\lambda_{r}^{\left[k\right]}\leq 1$
18: **else**
19: $\epsilon^{\left[k\right]}\leftarrow 1\;\;\;\;\;▷\;H_{0}\text{is rejected}.\;\lambda_{r}^{\left[k\right]}>1$
20: **end if**
21: **end for**
22: ${\hat{d}}_{r}=\text{count\_nonzero}(\epsilon )\;▷\;\text{Number of}\;\epsilon \;\text{entries equal}\;1$
**Output:** ${\hat{d}}_{r}$
2. The SCVs are resampled with replacement for B times, where for each resampling, V indices are drawn from ${𝒰}_{\text{int}}(1,V)$ (uniform distribution of integers between 1 and *V*) (line 4), and the SCVs are resampled on those indices (line 5). The resampled ${SCVs}_{b}{\hat{S}}_{r}$ thus also have V samples. The prescript b denotes the $b^{\text{th}}$ bootstrap resample.
3. The eigenvalues λ^rb from the covariance matrices C^rb=1VS^rbS^r⊤b are calculated (lines 6–7).
4. We define the test statistic $\hat{T}={\hat{\lambda }}_{r}^{\left[k\right]}-1$ (line 10).
5. The test statistic T^*b=λ^r[k]b−λ^r[k] is calculated (line 12).
6. The values of T^*b are sorted in ascending order (line 14). Using a given false alarm probability $P_{fa}$, a threshold T^*η is found, where $\eta =\left\lfloor (B+1)\cdot \left(1-P_{fa}\right)\right\rfloor$ (line 15), and T^*η is T^*b for b=η. If T^>T^*η, then $H_{0}$ is rejected, i.e., $\lambda_{r}^{\left[k\right]}$ is greater than 1 with a significance of $1-P_{fa}$, and the $k^{\text{th}}$ element in a vector ϵ is set to 1 (lines 16–20).
7. In the end, the number of 1s in ϵ is counted, which equals to ${\hat{d}}_{r}$, the estimated number of eigenvalues greater than 1 in the $r^{\text{th}}$ SCV (line 22).

### C. STEP 3: IDENTIFICATION OF THE RELATIONSHIP STRUCTURE USING STRUCTURED SCVS

In Step 3, the eigenvectors of the covariance matrices of the structured SCVs are used as features for the hierarchical clustering. In [20], it is shown that the eigenvector corresponding to an eigenvalue greater than 1 characterizes the correlated datasets for the corresponding block. More specifically, for each eigenvalue greater than 1, the eigenvector element corresponding to a dataset that is not part of the correlated datasets is 0, while the eigenvector elements of the correlated datasets are greater than 0. This means, the $d_{r}$ leading eigenvectors contain information about the relationship of the datasets within the $r^{\text{th}}$ SCV. As stated in Step 2, the covariance matrices are estimated from finite samples, and $d_{r}$ is also estimating using the proposed bootstrap-based hypothesis test. We will thus use this estimated ${\hat{d}}_{r}$ from Step 2. As different SCVs provide complementary information about the relationship of the datasets, the ${\hat{d}}_{r}$ leading eigenvectors (r∈𝒮) from all structured SCV covariance matrices are horizontally concatenated to form a feature matrix, F, of $\sum_{r\in 𝒮}{\hat{d}}_{r}$ eigenvector columns, which is then fed into the hierarchical clustering:

(13) $$F=\text{concat}\left({\hat{U}}_{i_{1}}\left(:,1\;:\;{\hat{d}}_{i_{1}}\right),\ldots ,{\hat{U}}_{i_{\left|𝒮\right|}}\left(:,\;1\;:\;{\hat{d}}_{i_{\left|𝒮\right|}}\right)\right)\in {\mathbb{R}}^{K\times \sum_{r\in 𝒮}{\hat{d}}_{r}}.$$

This way, our method leverages the knowledge of all SCVs together instead of performing an analysis separately on each SCV. The advantage of using hierarchical clustering, compared with, e.g., K-means clustering, is that no prior knowledge or estimation of the number of clusters is necessary. Additionally, while K-means clustering would only identify the groupings of datasets, which correspond to the resulting clusters, hierarchical clustering also identifies the relationship structure among the datasets, which is revealed in the dendrogram.

### D. COMPUTATIONAL COMPLEXITY

We compute the big-𝒪 complexity for each step of the proposed method. In Step 1, the complexity is dominated by the multiplication of ${\hat{C}}_{r}^{-1}\in {\mathbb{R}}^{K\times K}$ with ${\hat{S}}_{r}\in {\mathbb{R}}^{K\times V}$ in the main loop of IVA-L-SOS. In each iteration, ${\hat{C}}_{r}^{-1}$ is updated K times per SCV (and thus this multiplication is performed K times per SCV), which results for all R SCVs in a complexity of $𝒪\left(IRK^{3}V\right)$ for I iterations. In Step 2, the dominant cost is the calculation of the covariance matrices of the resampled SCVs S^rb∈ℝK×V, which for all B bootstrap resamples in all R SCVs has complexity $𝒪\left(BRK^{2}V\right)$. In Step 3, the cost is dominated by the hierarchical clustering of the K datasets, which is of complexity $𝒪\left(K^{3}\right)$ [25]. Thus, our method has a big-𝒪 complexity of $𝒪\left(RK^{2}V(IK+B)\right)$.

## IV. SIMULATIONS

**FIGURE 2.**
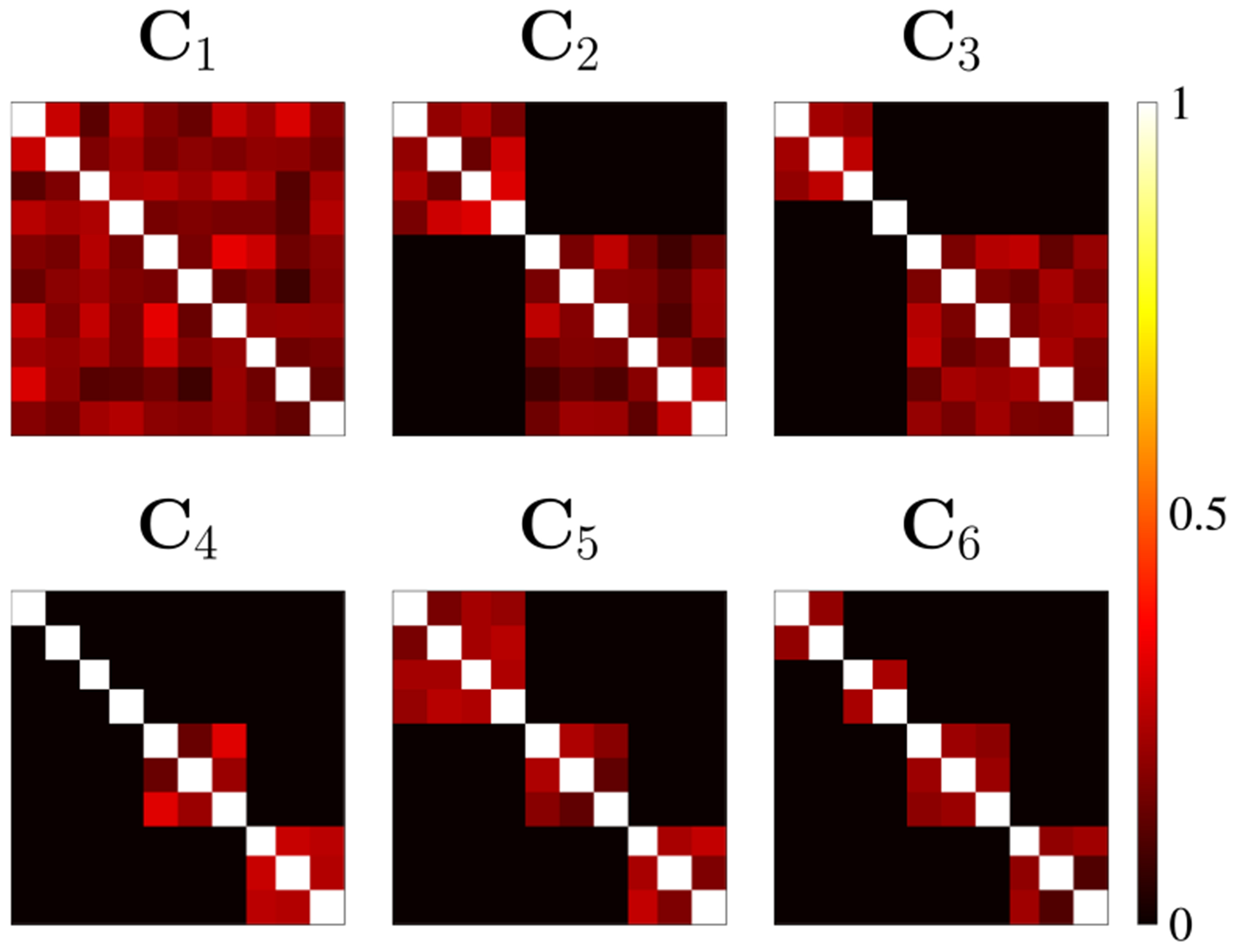
R=6 simulated SCV covariance matrices (of dimension 10×10) for ρ=0.2. We have $d_{1}=1,d_{2}=d_{3}=d_{4}=2,d_{5}=3$, and $d_{6}=4$. Using these covariance matrices, 6 SCVs with Laplacian-distributed sources are generated.

To demonstrate the performance of our proposed bootstrap technique, we simulate common and structured SCVs.^1^ We generate the entries of the SCV covariance matrices $C_{r}$ according to the following model:

(14) $$C_{r}(k,l)=\left\{\begin{array}{ll} 1, & k=l\;\text{(diagonal elements)}\\ 0, & k\neq l,C_{r}(k,l)\notin B_{i},1\leq i\leq d_{r}\\ \rho +n_{r}(k,l) & k\neq l,C_{r}(k,l)\in B_{i},1\leq i\leq d_{r} \end{array}\right.$$

where $B_{i}$ is the $i^{\text{th}}$ block, ρ is the correlation coefficient of the correlated sources, and $n_{r}(k,l)=n_{r}(l,k)∼𝒩(0, 0.0025)$ is added variability to fulfill the identifiability conditions of IVA [9]. Figure 2 shows the structure of the six 10×10 SCV covariance matrices in our simulations for ρ=0.2. $C_{1}$ corresponds to a common SCV, i.e., $d_{1}=1$, while the other SCVs are structured. $C_{2},C_{3}$ and $C_{4}$ all have two blocks, i.e., $d_{2}=d_{3}=d_{4}=2,C_{5}$ has 3 blocks, i.e., $d_{5}=3$, and $C_{6}$ has 4 blocks, i.e., $d_{6}=4$. Note that $C_{3}$ and $C_{4}$ contain 1 and 4 uncorrelated source components, respectively. Using these SCV covariance matrices, we generate R=6 SCVs $S_{r}\in {\mathbb{R}}^{K\times V}$, each with K=10 source components and V=1000 samples. The samples are drawn from a Laplacian distribution as described in [26] (in section 6.4), with zero mean and with covariance matrices specified as shown in Figure 2.

We perform two experiments. In the first (section IV-A), we compare the performance of our proposed bootstrap technique and two competing methods for estimating ${\hat{d}}_{r}$ from the true generated SCVs. We use the true sources here because the identified sources from IVA may be estimated correctly but not aligned correctly among SCVs, i.e., the correlated blocks in the covariance matrices may be permuted among SCVs. Due to this, ${\hat{d}}_{r}$ of the output of IVA may not match ${\hat{d}}_{r}$ of the generated sources. In a second experiment (section IV-B), we apply the complete method, including the IVA step and the clustering, on simulated observed datasets and investigate its robustness for different correlation coefficients. In both of these scenarios, we did not consider noise as we assumed that for real data, noise is effectively removed during a PCA-based dimension reduction step in the preprocessing. This is based on the assumption that the problem is essentially overdetermined, i.e., there are more observations than underlying source components of interest, which is the common scenario in most applications including ours.

### A. ESTIMATING ${\overset{ˆ}{d}}_{r}$

We evaluate the performance of three techniques for estimating ${\hat{d}}_{r}$ from the true sources. The first method is our proposed bootstrap (BT) technique, described in Algorithm 1, with B=1000 bootstrap resamples and $P_{\mathrm{fa}}=0.05$. The second method (EV) directly counts how many eigenvalues are greater than 1 in the $r^{\text{th}}$ SCV, i.e., ${\hat{d}}_{r}=\left|\left\{k\;:\;\lambda_{r}^{\left[k\right]}>1\right\}\right|$. The third method is the Gershgorin Disc (GD) technique from [16].

We simulate 50 Monte-Carlo runs. We investigate the two performance metrics

(15) $$P\left({\hat{d}}_{r}=d_{r}\right)=\frac{\#\left({\hat{d}}_{r}=d_{r}\right)}{\#\text{runs}},$$

which estimates the probability that ${\hat{d}}_{r}$ is estimated correctly, and $\mu_{{\hat{d}}_{r}}$, which is the average value of the estimated ${\hat{d}}_{r}$.

**FIGURE 3.**
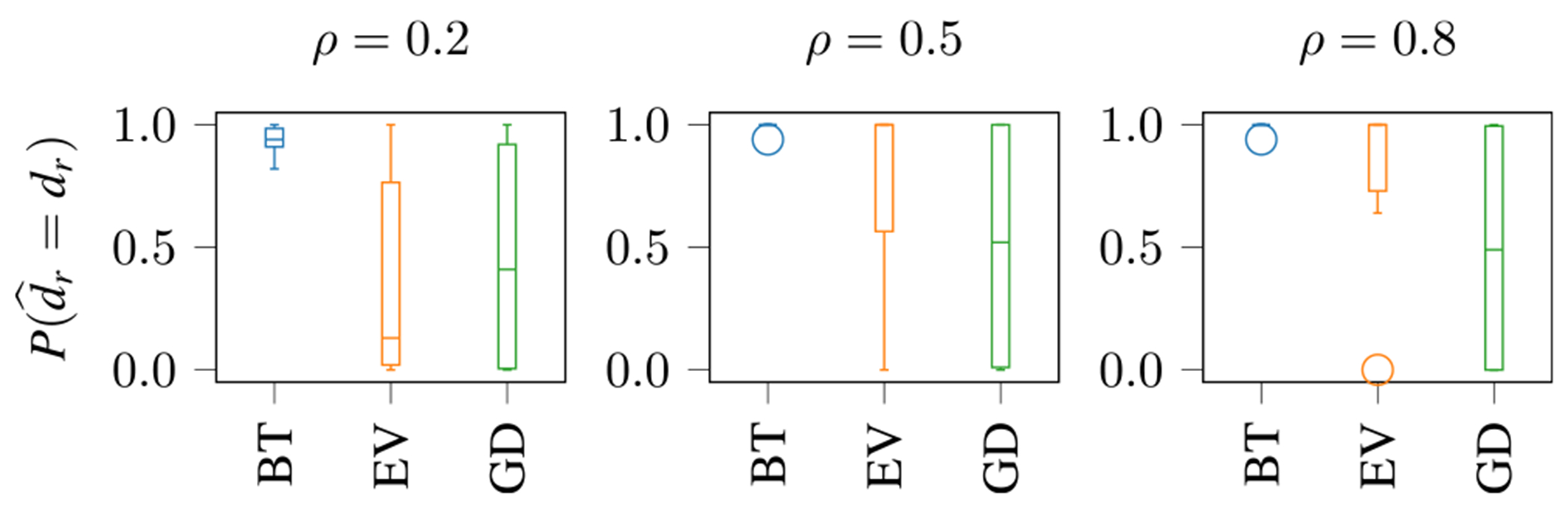
Boxplot of $P\left({\hat{d}}_{r}=d_{r}\right),r=1,\ldots ,6$, for different values of ρ for the BT, EV and GD technique. On average, BT is always superior to EV and GD.

The boxplot of $P\left({\hat{d}}_{r}=d_{r}\right)$ for the R=6SCVs is shown in Figure 3 for different values of ρ, with circles denoting outliers. Notably, our proposed BT technique demonstrates robust performance by accurately estimating ${\hat{d}}_{r}$ even for very small correlation values in the underlying data, showcasing its effectiveness in handling the Laplacian (non-Gaussian) data distribution and varying correlation values ρ. In contrast, the EV technique only achieves high $P\left({\hat{d}}_{r}=d_{r}\right)$ values with increasing ρ, while the GD technique does not perform well for all values of ρ.

**FIGURE 4.**
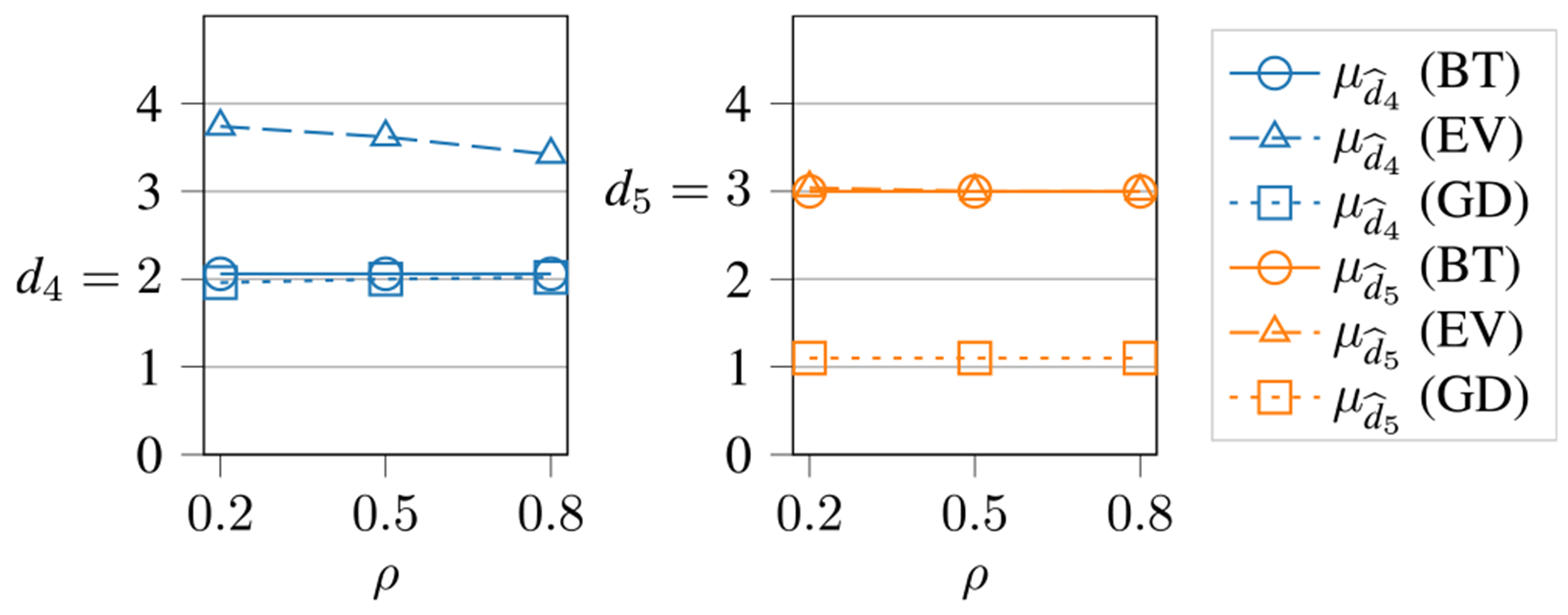
Average value of ${\overset{ˆ}{d}}_{r}$ for SCVs 4 and 5 for different values of ρ for the BT, EV and GD technique. The true values are $d_{4}=2$ and $d_{5}=3$. The BT technique estimates both values close to the ground truth.

To investigate the reason for small values of $P\left({\hat{d}}_{r}=d_{r}\right)$ in the EV and GD technique, we show $\mu_{{\hat{d}}_{4}}$ and $\mu_{{\hat{d}}_{5}}$ in Figure 4, estimated for the BT, EV and GD techniques. We see (blue triangles) that the EV technique overestimates $d_{4}$ (also $d_{3}$, not shown here), with an improvement for higher ρ values, while the BT (circles) and GD (squares) techniques estimate the value close to the ground truth for all ρ values. In orange squares, we see that the GD technique strongly underestimates $d_{5}$ (also $d_{2}$ and $d_{6}$, not shown) compared with the BT (circles) and EV (triangles) techniques for all ρ values. The over- and underestimation of ${\hat{d}}_{r}$ in the EV and GD techniques is the reason for the decreased values of $P\left({\hat{d}}_{r}=d_{r}\right)$ compared with the BT technique. Thus, the BT technique is superior to EV and GD in terms of estimating ${\hat{d}}_{r}$.

### B. IDENTIFYING THE RELATIONSHIP STRUCTURE

As we have shown that the BT technique is superior to EV and GD in terms of estimating ${\hat{d}}_{r}$, we evaluate our complete method for identifying the relationship structure only using BT. We are not aware of other methods to identify the relationship structure, and not only groupings of the datasets, with whom we could compare our method.

We generate the SCVs as in section IV-A and the datasets as $X^{\left[k\right]}=A^{\left[k\right]}S^{\left[k\right]}$, where the elements of $A^{\left[k\right]}\in {\mathbb{R}}^{6\times 6}$ are drawn from 𝒩(0,1). After performing IVA-L-SOS with 20 random initializations and choosing the estimated sources of the most consistent run^2^ as in [27], the BT technique is applied to the estimated SCVs to estimate ${\hat{d}}_{r}$. The ${\hat{d}}_{r}$ leading eigenvectors of the structured SCVs are concatenated in a feature matrix F, which we feed into the hierarchical clustering. Hierarchical clustering is performed using the linkage function from scikit-learn [28] with the ‘ward’ linkage (minimizing the variance of the clusters) and visualized using the dendrogram function.

**FIGURE 5.**
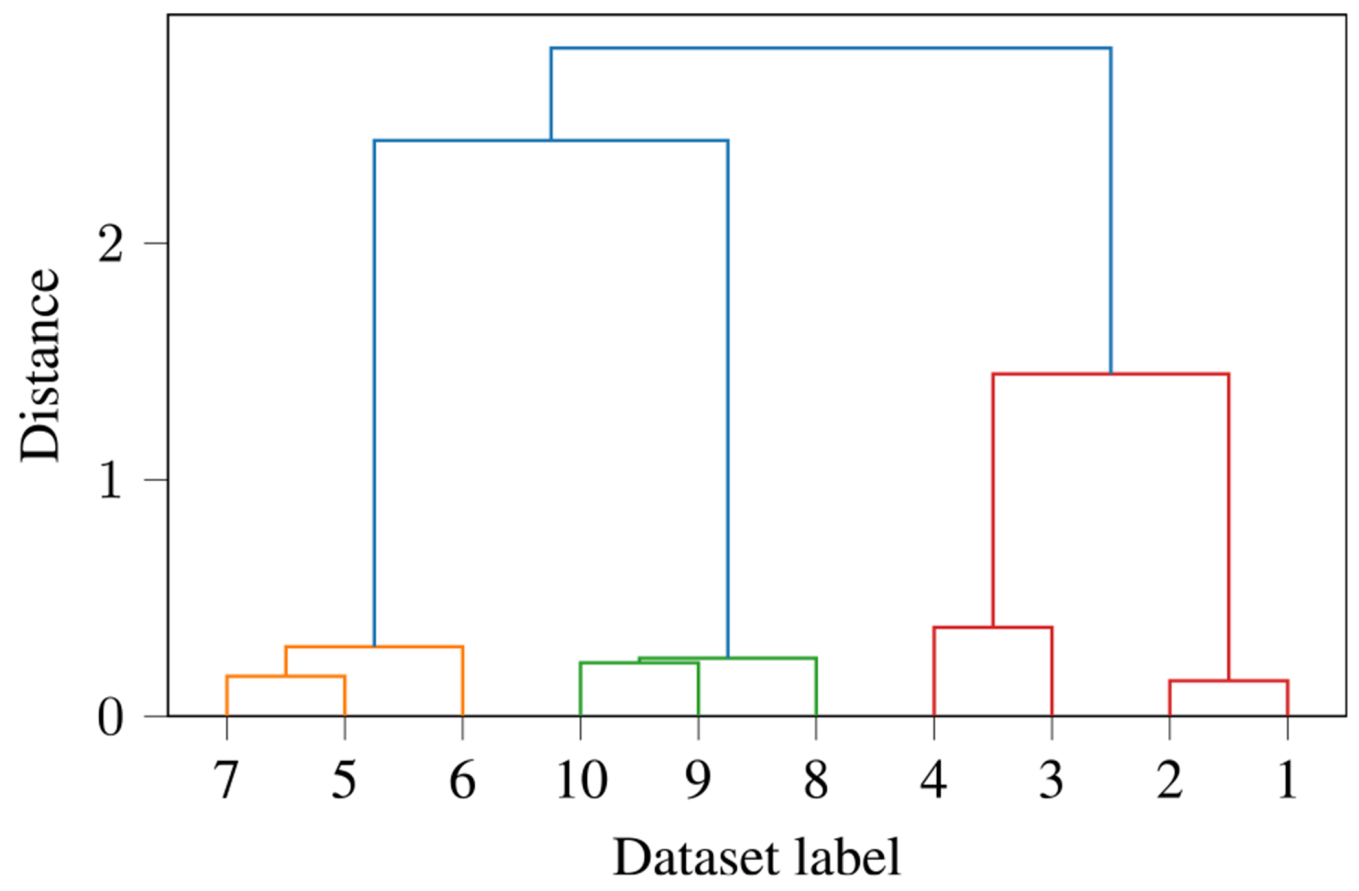
The ground truth dendrogram for our simulations shows the relationship structure of the 10 datasets. There exist 3 groupings: consisting of datasets 5–7 (orange), 8–10 (green), and 1–4 (red).

Figure 5 shows the ground truth of the dendrogram for clustering the datasets using the SCV covariance matrices of this experiment. Ground truth means that we set $d_{r}$ manually and use the $d_{r}$ eigenvectors of the true (instead of sample) covariance matrix for clustering. In the figure, the labels of the datasets are denoted on the x-axis, and the y-axis shows the distance between the clusters. A small distance means that the corresponding clusters are very similar. We see that three groupings exist in the datasets, i.e., datasets 5–7 form one grouping (orange), datasets 8–10 form a second grouping (green), and datasets 1–4 form a third grouping (red). We also see that the groupings of datasets 5–7 and 8–10 are closer to each other than to that of datasets 1–4. The labels that result from the clustering are the same within each cluster but with arbitrary ordering. By denoting the true label for the $k^{\text{th}}$ dataset with $c_{k}$, we choose $c_{1}=c_{2}=c_{3}=c_{4}=1$, $c_{5}=c_{6}=c_{7}=2$, and $c_{8}=c_{9}=c_{10}=3$.

We are not aware of a performance metric that captures how well the relationship structure of the datasets is estimated; instead, we use the Adjusted Mutual Information (AMI) [29] between the true and estimated clusters, which evaluates if the groupings are identified correctly. The AMI is a normalized metric based on the mutual information of the true and estimated clustering, i.e., the AMI is equal to 1 if the true and the estimated clusters are equal, and equal to 0 if the mutual information between the true and estimated clusters equals the expected value of the mutual information between the true and a random clustering. Furthermore, the AMI corrects for the permutation ambiguity between the true and the estimated clusters. Our method has an AMI of 1 for ρ=0.2, 0.5, 0.8, i.e., it correctly identifies the groupings in 100% of the runs for all the correlation values.

## V. REAL FMRI MULTI-TASK DATA

Finally, we apply our method on real multi-task fMRI data. Data from multiple tasks provide complementary information about the brain [30], [31] because different tasks involve cognitive functions that are either task-specific or common across all tasks [32]. By analyzing multiple datasets jointly, the function of and relationship between brain networks can be identified [32], which helps to understand the brain organization [33]. Furthermore, jointly analyzing data from multiple cognitive tasks may also help to understand complex disorders like schizophrenia, which is a neuropsychiatric disorder associated with cognitive deficits [30], [34] and altered connections between brain regions [35]. These altered connections might not be captured when analyzing only the data from a single task [30]. By analyzing data from multiple tasks jointly, latent neural patterns, i.e., biomarkers, may be revealed, which help yield new features like a common network for the tasks that captures differences between patients with schizophrenia and controls [11], [35].

### A. DATASETS AND PREPROCESSING

We apply our proposed method on 10 fMRI datasets from the MIND Clinical Imaging Consortium (MCIC) collection [36], which are collected from 271 subjects (121 patients with schizophrenia and 150 healthy controls) that perform three different tasks: Auditory Oddball (AOD), Sensory Motor (SM), and Sternberg Item Recognition Paradigm (SIRP). In the AOD task, three stimuli are played: a frequent standard stimulus (1 kHz tone), an infrequent target stimulus (1.2 kHz tone), and an infrequent novel stimulus (computer-generated, complex sound). After hearing the target stimulus, the subject must press a button with the right index finger. During the SM task, tones are played in increasing order until the highest pitch is reached, then in decreasing order. The subject must press a button with the right thumb every time a new tone occurs. The SIRP task consists of two phases, encoding (SIRP-E) and probe (SIRP-P). In the encoding phase, a set of digits is presented on a screen, and the subject needs to learn this set. In the following probe (SIRP-P), digits are presented subsequently in a pseudo-random order. The subject must press a button with the right thumb if the digit was in the set and with the left thumb if not.

We expect task-specific activations of the auditory brain regions for the auditory tasks (AOD and SM) and of the visual brain regions for the visual tasks (SIRP), along with activations of the Default Mode Network (DMN) for all tasks. This prior knowledge about the relationship of the task datasets provides us with the opportunity to directly assess the success of the proposed method with real data, which is typically not easily achievable, as for real data one often does not know the true relationship structure. Furthermore, as the data is collected from patients and controls, we can also use these datasets to evaluate which tasks show a clearer discrimination of patients with schizophrenia.

For each of the subjects, multiple three-dimensional brain scans are collected over time during each task. The recorded scans are then preprocessed as in [35]: Using the Statistical Parametric Mapping (SPM) MATLAB toolbox [37], a simple voxelwise linear regression is applied to the data to eliminate the temporal dimension. The regressors are created by convolving the hemodynamic response function in SPM with the desired predictors for each task, which will be described in the next paragraph. For each subject and task, the resulting regression coefficient maps, also called “contrast images”, are flattened and used as one-dimensional features that capture the variations across subjects. The flattened feature vectors of length J=48, 546 voxels are concatenated for I=271 subjects to create the $k^{\text{th}}$ task dataset $X^{\left[k\right]}\in {\mathbb{R}}^{I\times J}$, k=1,…,K.

In the AOD task, the occurrences of the novel stimuli (AOD-N), the target stimuli (AOD-T), and the target with standard stimuli (AOD-TS) are each modeled as delta functions and used as predictors. Thus, there are three task datasets for the AOD task. In the SM task, the whole block is used as the predictor; thus, there is one SM task dataset. For both phases of SIRP (E and P), also the whole block is used as the predictor. This way, the data allows us to analyze the learning and retrieving phases of this task separately. As the SIRP task is repeated with 1, 3, and 5 digits in the set, there are six datasets for the SIRP task. Thus, in total, we have K=10 datasets.

### B. IMPLEMENTATION DETAILS

As typically the dimension of the datasets is much higher than the number of latent sources, a dimension reduction via Principal Component Analysis (PCA) is performed as pre-processing. This way, each observed dataset is transformed separately in a lower-dimensional subspace of dimension R. Then, IVA is performed on the dimension-reduced datasets $X^{\left[k\right]}\in {\mathbb{R}}^{R\times V}$ to estimate the source matrices ${\hat{S}}^{\left[k\right]}\in {\mathbb{R}}^{R\times V}$. From these, the SCVs ${\hat{S}}_{r},r=1,\ldots ,R$ are formed.

As it is mostly the case for real data, also in the MCIC data, we do not know the ground truth dimension R of the latent sources. Thus, selecting an appropriate value for R is important to get meaningful results. Our method gives robust clustering results for a wide range of values for R=20,21,…,30. In this paper, we present the results for R=25 because at this order, the estimated fMRI activation maps are 1) stable, i.e., not split (as in higher orders) or merged (as in lower orders), and 2) meaningful, i.e., physically interpretable.

Our approach is primarily data-driven, with the exception of two user-selected parameters, the probability of false alarm $P_{fa}$ and the number of bootstrap resamples B. With $P_{fa}$, we can directly control the risk of overestimating $d_{r}$, i.e., the number of eigenvalues greater than 1 in the $r^{\text{th}}SCV$ covariance matrix. The higher the value for B is chosen, the better the distribution is estimated, but for a too high value of B, there will not be a better estimate at some point. We choose B=1000 bootstrap resamples and $P_{fa}=0.05$ to estimate ${\hat{d}}_{r}$, as these are typical values for these parameters and achieve good results in general.

As we are also interested in identifying the components that discriminate between patients and controls, we apply a two-sample t-test on the first 150 (controls) and the following 121 (patients) values of each column of the estimated mixing matrix ${\hat{A}}^{\left[k\right]}$. We consider p-values smaller than 0.05 to be significant, indicating that the corresponding activated brain areas are different in patients with schizophrenia and healthy controls. Using this t-test, we corrected the signs of the estimated sources ${\hat{S}}^{\left[k\right]}$ to overcome the sign ambiguity of IVA: We made sure that the t-values of the datasets that show a significant difference between patients and controls (p<0.05) are positive or made positive by multiplying the estimated sources and corresponding subject profiles by −1 (if the t-value is negative). This way, positive values of the (zero-mean) sources indicate higher activations in controls, and negative values indicate higher activation in patients.

### C. RESULTS WITH THE fMRI DATA

#### 1) IDENTIFICATION OF COMMON AND STRUCTURED SCVs

**FIGURE 6.**
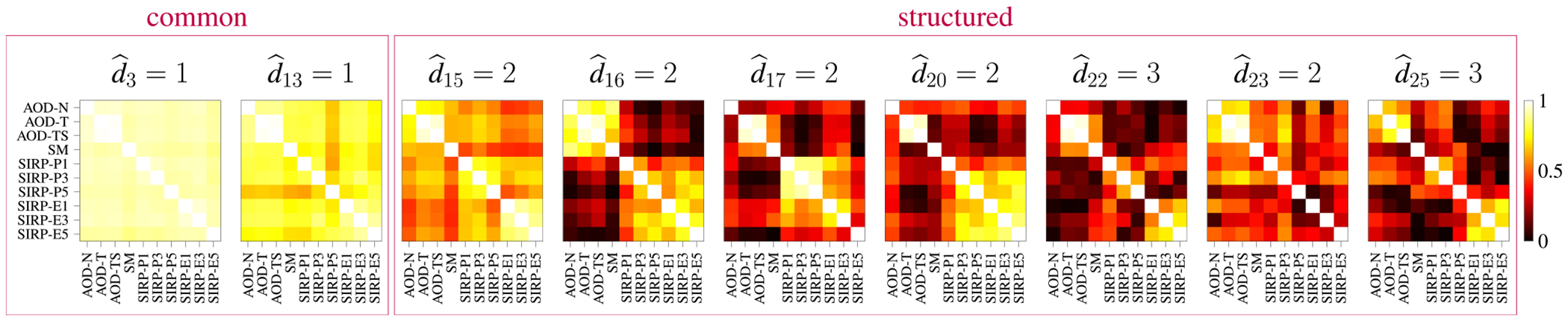
Subset of the estimated SCV covariance matrices. SCVs 3 and 13 are identified as common, and SCVs 15–17, 20, 22–23, and 25 are identified as structured.

In Figure 6, a subset of the covariance matrices of the SCVs estimated by IVA-L-SOS is shown along with the estimated values for ${\hat{d}}_{r}$. Light values correspond to high correlations. For SCVs 3 and 13, ${\hat{d}}_{r}=1$, thus, these SCVs are identified as common. SCVs 15–17, 20, 22–23, and 25 are identified as structured because ${\hat{d}}_{r}>1$. The visible block structure in each SCV already reveals information about the relationship of the datasets [14]. For example, the covariance matrix of SCV16 clearly shows high correlations within the AOD and SM tasks and within the SIRP tasks, but small correlations across those tasks.

#### 2) IVA-L-SOS

The estimated source components (fMRI activation maps) provide information about which brain regions are activated in which tasks. In the following, we present SCV3, SCV16, and SCV17 as examples for common and structured SCVs, because they either show activations in brain areas that are common in all tasks or correspond to the specific tasks. All fMRI activation maps are thresholded with |z|=2 before visualization and plotted above the structural anatomical images. Significant p-values are displayed in magenta with the superscript *. Because of the sign correction, red or yellow voxels indicate that a brain area is activated higher in controls, and blue voxels indicate higher activation in patients. Then, we corrected the sign of the non-significant datasets manually by matching the color (red/blue) of the activated areas to the significant datasets of the same task.

**FIGURE 7.**
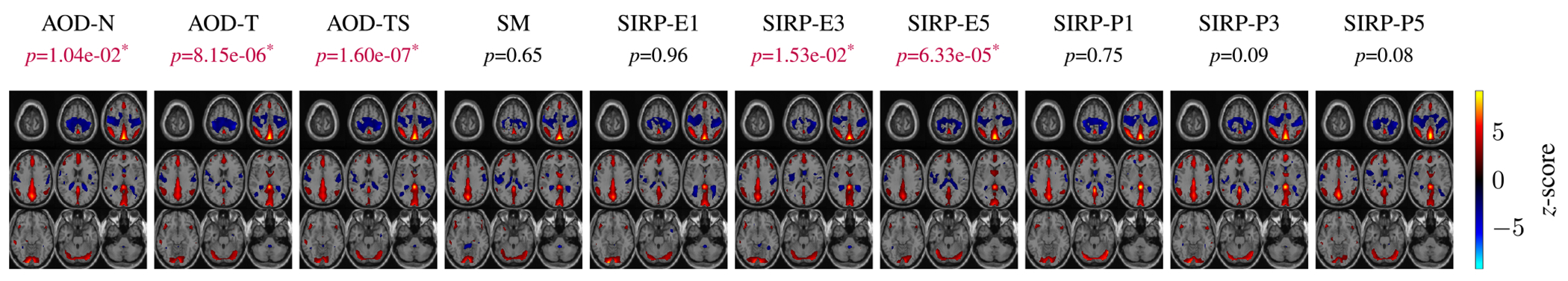
Estimated fMRI activation maps corresponding to SCV3. The default mode network and the visual regions are activated higher in controls (shown by red/yellow voxels), while the sensorimotor areas are activated higher in patients (shown by blue voxels).

In SCV 3 in Figure 7, we see activations of the DMN (red areas in slices 4–6). The DMN is known to have a decreased activation when a task is performed [32], [38]. The higher activation of the DMN in controls means that the deactivation is stronger in patients. This can be interpreted as patients needing to focus more on a task to perform it well. The sensorimotor regions (blue areas in slices 2–3) are activated higher in patients and therefore support this interpretation. There are minor activations in the visual regions (red areas in slices 7–9), which are expected because the subjects had their eyes open during all tasks. The very small p-values in the AOD-T and AOD-TS datasets indicate that especially when the target stimulus occurs, the patients are significantly stronger engaged with the task, i.e., have significantly smaller activation of the DMN. These lower p-values in the AOD datasets are expected since the AOD task has been shown to be important in discriminating patients with schizophrenia and healthy controls, as patients have a smaller oddball response [39]. In the SIRP-P datasets, there is no significant difference between patients and controls for the DMN. In contrast, in the SIRP-E datasets, the p-values become smaller with increasing task difficulty, i.e., the deactivations of the DMN become stronger for the patients. This coincides with the literature, as with an increasing level of difficulty of a task, the deactivation of the DMN becomes stronger [32].

**FIGURE 8.**
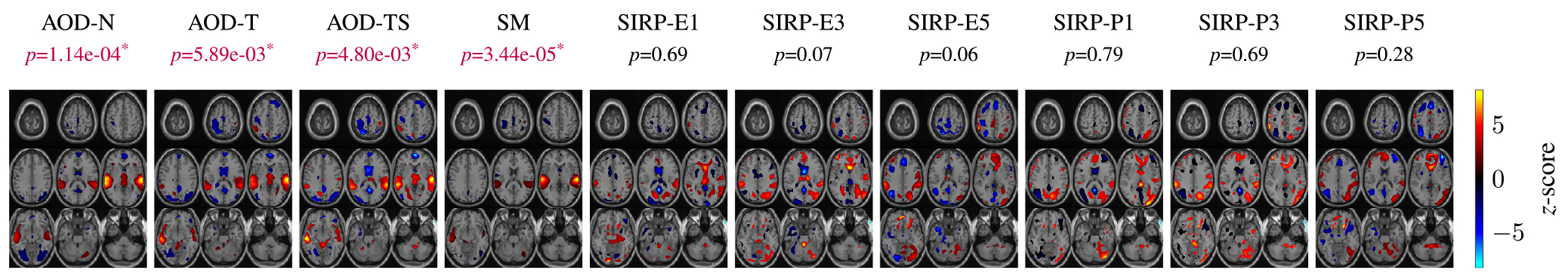
Estimated fMRI activation maps corresponding to SCV16. The auditory regions are activated higher in controls in the AOD and SM datasets and not activated in the SIRP datasets.

The fMRI activation maps corresponding to SCV16 are shown in Figure 8. The auditory regions (red areas in slices 5–7) are activated higher in the controls in the AOD and SM datasets. The p-values are also significant for the AOD and SM datasets, supporting the literature that activations in the auditory regions may be a biomarker for differentiating patients with schizophrenia and healthy controls [40].

**FIGURE 9.**
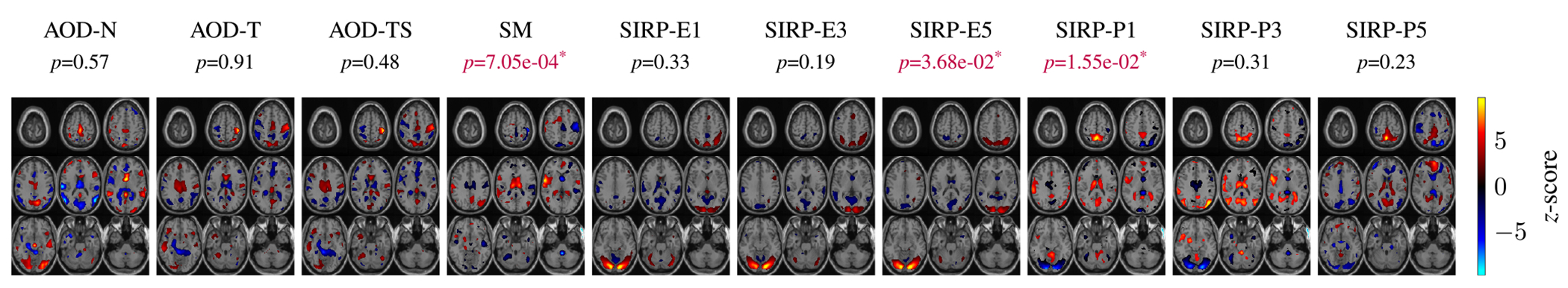
Estimated fMRI activation maps corresponding to SCV17. The visual regions are activated higher in controls in the SIRP-E datasets, higher in patients in the SIRP-P datasets, and not activated in the AOD and SM datasets.

In contrast, the fMRI activation maps corresponding to SCV17, shown in Figure 9, show strong activations in the visual areas (red/blue areas in slice 7) for the SIRP datasets. The significant difference between patients and controls here is found for SIRP-E5 (in accordance with SCV3) and SIRP-P1. What is most surprising here is that visual regions are activated higher in controls for the SIRP encoding but higher in patients for the SIRP probe. An explanation might be that patients need to focus more on a digit to remember it, while the controls just briefly see the digit, and their memory can be accessed faster.

#### 3) IDENTIFICATION OF THE RELATIONSHIP STRUCTURE

**FIGURE 10.**
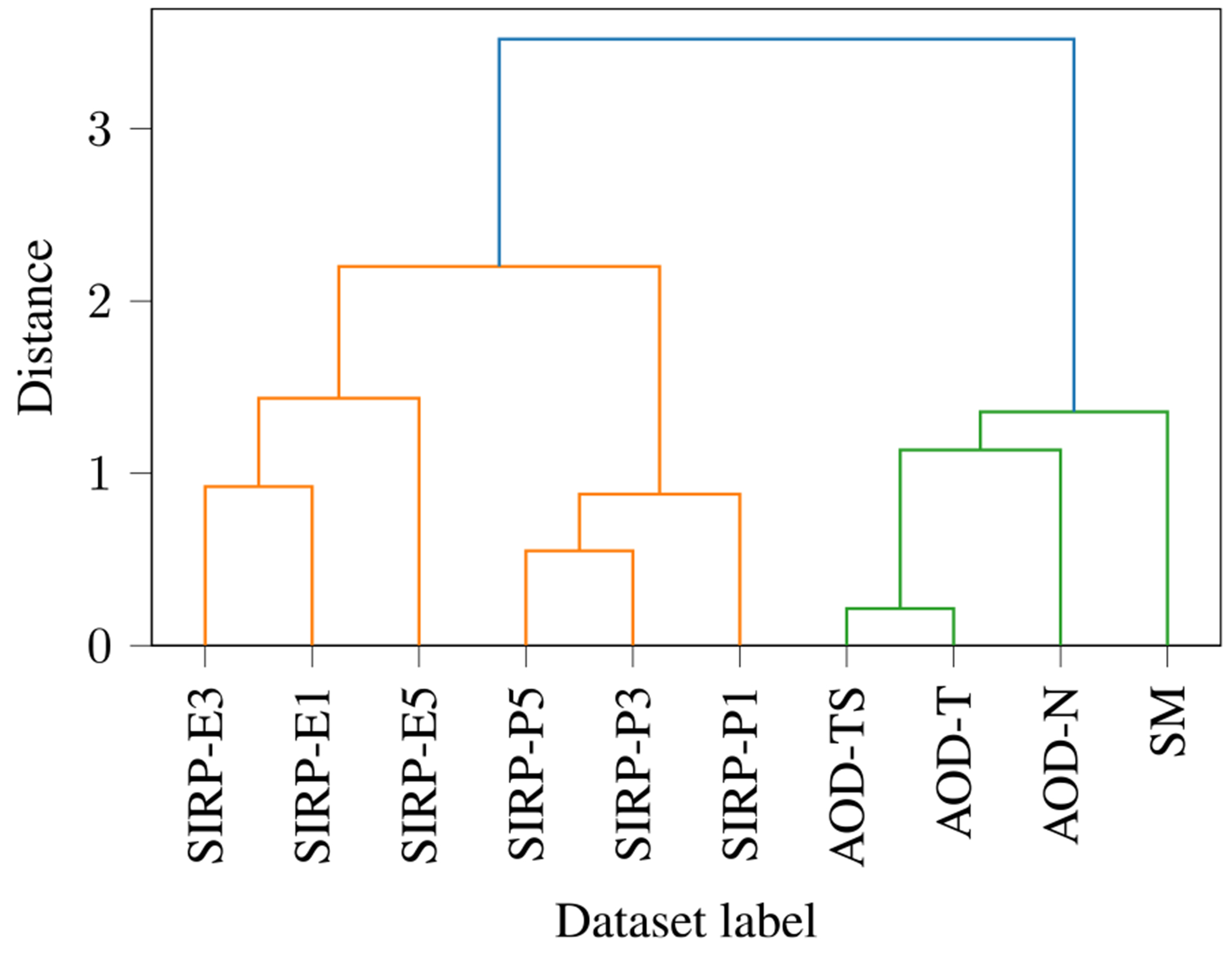
The dendrogram for the real data reveals the relationship structure among the datasets. Two groupings are found, consisting of the SIRP datasets (orange) and of the auditory datasets, AOD and SM (green). Within the SIRP datasets, the SIRP-E and SIRP-P datasets form two groupings. Within the auditory datasets, there is one grouping consisting of the AOD datasets.

The information of all SCVs is fused to identify the relationship structure among the datasets. As described in section III-C, the ${\hat{d}}_{r}$ leading eigenvectors of the structured SCVs are concatenated to form a feature matrix, which is the input of the clustering algorithm. The dendrogram for the hierarchical clustering of the feature matrix is shown in Figure 10. Here, a grouping refers to a group of task datasets (in contrast to a group of subjects, as in subgroup identification). The SIRP tasks form one grouping (orange), and the AOD and SM tasks form a second grouping (green). As the SIRP task involves a visual stimulus and the AOD and SM tasks both involve auditory stimuli, these resulting groupings are meaningful. Within the SIRP grouping, there are two finer groupings visible: one consisting of the encoding datasets and one the other of the probe datasets. This makes sense because they refer to two different phases of the SIRP task. Within the auditory grouping, the AOD datasets form another grouping.

## VI. DISCUSSION

In this paper, we have proposed a method to identify the relationship structure among multiple datasets. Our method consists of three steps: 1) estimating latent sources from observed datasets using IVA, 2) identifying structured SCVs (SCVs whose covariance matrices have more than one eigenvalue greater than 1), and 3) extracting features from the structured SCVs, which are then used by hierarchical clustering to identify the relationship structure among the datasets. Compared with previous studies, our proposed method alleviates the need to assume Gaussianity in the data by 1) including higher-order statistics through the use of IVA-L-SOS for source estimation, which leads to more interpretable components, and 2) not relying on any distributional assumptions, which is achieved by estimating the number of eigenvalues greater than 1 using their theoretical properties and a bootstrap-based hypothesis testing approach.

Our simulations demonstrate the success of our method in terms of 1) correctly estimating the number of blocks per SCV against competing methods and 2) identifying the relationship structure among multiple datasets. Applying our method to real multi-task fMRI data has revealed activated brain areas that are known to be affected by schizophrenia: We see significantly stronger deactivations of the DMN in patients and significantly stronger activations of the auditory brain regions in controls. The identified relationship structure of the task datasets is consistent with, but extends, existing work. While we were able to draw conclusions from the here presented estimated fMRI maps, it is important to note that interpretation is not always straightforward. We have carefully selected and presented SCVs that show activations in meaningful brain regions, which we identified according to Brodmann areas [41]. Despite the simplicity of the task design, many of the unrepresented components defy straightforward explanation. However, interpreting a subset of the most meaningful components can already help in understanding how the brain functions. Thus, a good guideline is to find a range of R values that lead to stable results after the dimension reduction and then go through the estimated components and compare them with established brain regions such as the Brodmann areas [41] to facilitate interpretation. It is important to remember that relying solely on comparisons with known brain atlas components might cause us to overlook brain regions not included in the user-defined atlas. Nevertheless, comparing the brain regions with an atlas provides an initial reference point before conducting further investigations.

Importantly, the proposed method is not limited to task datasets but is applicable to more general problems, e.g., for identifying subgroups of subjects in neuroimaging, and other fields. After having provided the confirmation of the success of our method on these clinically well-understood datasets, the direct implications for clinical significance and treatment strategies could be further explored in the future by applying our method to diverse datasets, with a specific focus on precision medicine and other relevant applications. For example, by identifying the relationship structure among patient datasets, our method allows for a detailed analysis of associations among data of, e.g., patients with schizophrenia, bipolar disorder, and their subtypes. However, a limitation of our proposed method is that it does not identify SCVs with identity covariance matrices, i.e., SCVs that consist of completely uncorrelated sources. A possible way to overcome this limitation in the future may be to adapt the bootstrap test.

## Data Availability

The IRB has determined the data cannot be shared outside the study team, and any ongoing use must be approved by the fBIRN PI.

## Footnotes

1 Python code available at https://github.com/SSTGroup/relationship_structure_identification

2 Python code available at https://github.com/SSTGroup/independent_vector_analysis [14]
